# Impact of real-life use of artificial intelligence as support for human reading in a population-based breast cancer screening program with mammography and tomosynthesis

**DOI:** 10.1007/s00330-023-10426-4

**Published:** 2023-11-17

**Authors:** Esperanza Elías-Cabot, Sara Romero-Martín, José Luis Raya-Povedano, A.-K. Brehl, Marina Álvarez-Benito

**Affiliations:** 1 Maimónides Biomedical Research Institute of Córdoba (IMIBIC), Córdoba, Spain; 2 Breast Cancer Unit, Department of Diagnostic Radiology, Reina Sofía University Hospital, Menéndez Pidal Avenue s/n, 14004 Córdoba, Spain; 3 University of Córdoba, Córdoba, Spain; 4 ScreenPoint Medical BV, Toernooiveld 300, 6525 EC Nijmegen, The Netherlands

**Keywords:** Artificial Intelligence, Mass screening, Mammography, Digital breast tomosynthesis, Breast neoplasms

## Abstract

**Objectives:**

To evaluate the impact of using an artificial intelligence (AI) system as support for human double reading in a real-life scenario of a breast cancer screening program with digital mammography (DM) or digital breast tomosynthesis (DBT).

**Material and methods:**

We analyzed the performance of double reading screening with mammography and tomosynthesis after implementarion of AI as decision support. The study group consisted of a consecutive cohort of 1 year screening between March 2021 and March 2022 where double reading was performed with concurrent AI support that automatically detects and highlights lesions suspicious of breast cancer in mammography and tomosynthesis. Screening performance was measured as cancer detection rate (CDR), recall rate (RR), and positive predictive value (PPV) of recalls. Performance in the study group was compared using a McNemar test to a control group that included a screening cohort of the same size, recorded just prior to the implementation of AI.

**Results:**

A total of 11,998 women (mean age 57.59 years ± 5.8 [sd]) were included in the study group (5049 DM and 6949 DBT). Comparing global results (including DM and DBT) of double reading with vs. without AI support, we observed an increase in CDR, PPV, and RR by 3.2/‰ (5.8 vs. 9; *p *< 0.001), 4% (10.6 vs. 14.6; *p *< 0.001), and 0.7% (5.4 vs. 6.1; *p *< 0.001) respectively.

**Conclusion:**

AI used as support for human double reading in a real-life breast cancer screening program with DM and DBT increases CDR and PPV of the recalled women.

**Clinical relevance statement:**

Artificial intelligence as support for human double reading improves accuracy in a real-life breast cancer screening program both in digital mammography and digital breast tomosynthesis.

**Key Points:**

*• AI systems based on deep learning technology offer potential for improving breast cancer screening programs.*

*• Using artificial intelligence as support for reading improves radiologists’ performance in breast cancer screening programs with mammography or tomosynthesis.*

• *Artificial intelligence used concurrently with human reading in clinical screening practice increases breast cancer detection rate and positive predictive value of the recalled women*.

## Introduction

Breast screening with mammography is considered the most effective method to decrease breast cancer mortality as it increases the chance for early detection [1, 2]. However, up to 24% of breast cancers are missed despite regular screening [3]. The high volume of mammograms to be read in screening programs, most of which will be normal, and the presentations of cancer as small subtle lesions, can reduce the performance of radiologists, increasing the risk of false negatives [4, 5].

Traditional computer-aided detection (CAD) systems, in the context of breast cancer screening, have not been shown to improve diagnostic accuracy [6] and have been associated with a significant increase in false positives and recall rates [7].

Recently, the development of new artificial intelligence (AI) systems based on “Deep learning” technology has improved traditional CAD algorithms and shows great potential in the field of breast imaging [8]. By providing large amounts of training data to a deep learning algorithm, deep learning inherently defines the optimal features that are necessary to detect suspicious areas. This is an advantage of neural network–based algorithms towards machine learning (i.e., random forest) [9, 10], where feature selection is not performed by the network itself but has been previously defined. Deep learning neural network–based algorithms allow for predictive analytics and improve their performance depending on the size of training data. In this way, these AI systems not only identify suspicious findings in the image, but also establishes an overall exam score indicating the overall cancer risk. This ability to stratify digital mammograms and tomosynthesis has been analyzed in retrospective simulated studies to reduce reading workload up to 70% without a negative impact in cancer detection [11–15]. In a recently published study conducted prospectively in a real-world screening setting, AI-assisted reading was associated with a significant reduction in screen-reading workload (44%), and similar cancer detection rate [16].

Deep learning–based AI systems implemented as a stand-alone solution have been shown to yield performance levels similar to the performance of a radiologist, with an AUC ranging from 0.7 to 0.96 [17–21].

Furthermore, AI systems can be used for concurrent clinical decision support during reading of DM or DBT exams. Many studies have shown that radiologists improved their cancer detection accuracy when using an AI system as concurrent reading support [22–27], while decreasing their false positive and recall rates [24, 25]. However, none of these studies have been carried out in real-world screening programs and have used cancer-enriched datasets. Such laboratory conditions might affect the reader’s behavior and are not fully transferable to clinical screening practices.

In this study, we evaluate a consecutive cohort of screening mammograms and tomosynthesis that were double read with concurrent AI support, in a real screening environment, assessing the impact it has had on detection and recall rates.

To the best of our knowledge, there are no studies that investigate this effect in a large and consecutive series, in the real setting of a population-based screening program.

## Material and methods

This study has been carried out in a single institution, the Córdoba Breast Cancer Screening Program (CBCSP), and did not receive any private or public funding. The authors who were in control of the data and information submitted for publication were not employees of or consultants for Screenpoint Medical (E.E.C., S.R.M., J.L.R.P. and M.A.B.). The requirement for informed consent was waived by the hospital's institutional review board.

The CBCSP is part of the Andalusian screening program in Spain. All women aged 50–69 are invited to participate with a biennial mammogram or tomosynthesis depending on resource availability.

In the CBCSP, all exams are independently double read, without consensus or arbitration (women are recalled if any reader decides to recall). The readings are performed according to Breast Imaging Reporting and Data System (BI-RADS) terminology. Studies classified as BI-RADS 1 and 2 are not recalled. Studies classified as BI-RADS 3, 4, and 5 are recalled and sent to the reference hospital. Women recalled are subjected to diagnostic workup (special mammography projections, tomosynthesis, contrast mammography, ultrasound, or MRI). Suspected findings are confirmed by percutaneous biopsy.

In March 2021, a commercially available breast cancer detection AI software was implemented in the CBCSP to assist the screening radiologists with mammography and tomosynthesis readings.

This study evaluates screening performance in the consecutive screening cohort 1 year after implementation of AI, the study group, and compares to a control group of the same size just prior to the implementation of AI..

### Study group (with AI support)

**Fig. 1:**
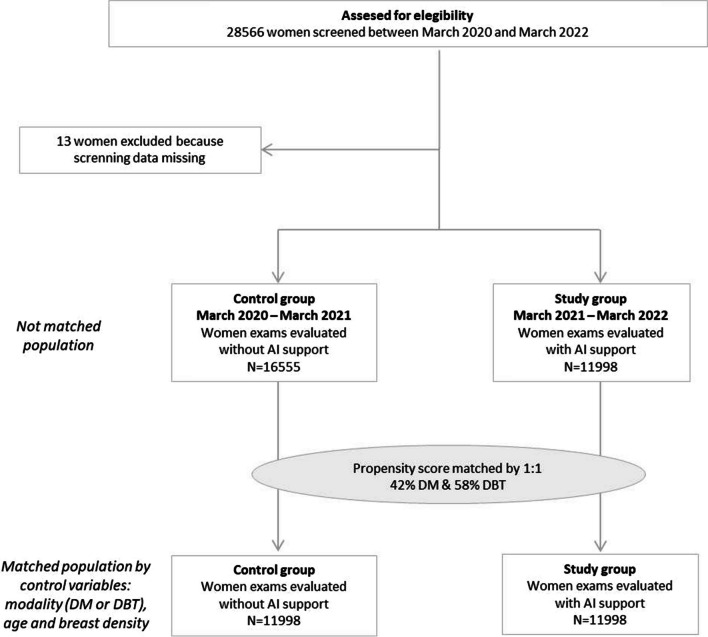
Study design

The study group consisted of women (age range, 50–69) who were consecutively screened with digital mammography (DM) or digital breast tomosynthesis (DBT), between 15 March 2021 and 14 March 2022, using a Selenia device (Hologic) for DM and a Selenia Dimensions device (Hologic) for DBT, respectively. We excluded recalled women whose diagnostic workup results were not accessible (Fig. 1).

During this period, all exams were double read in the screening program, by a team of ten radiologists dedicated to breast imaging (2 to 20 years of experience in breast imaging at the time of the study), concurrently using an AI system for cancer detection support. The radiologists read the DM and DBT exams while viewing the AI system findings. The final decision on whether or not to recall was made by the radiologist.

### AI support system

The AI system used in this study was the commercially available AI system Transpara®, (version 1.7.0, ScreenPoint Medical) which has received Conformité Européenne (CE) mark approval and was cleared by the U.S Food and Drug Administration. This software has been investigated in other publications [11, 12, 14, 17, 18, 22, 23, 27].

The AI system is based on deep convolutional neural networks and has been trained, validated, and tested on more than 1 million mammograms, to detect suspicious soft tissue lesions and calcifications on digital mammograms and tomosynthesis across machines from different vendors. These lesions are marked with a score from 1 to 100 according to the probability of malignancy. Based on the lesion detected with the highest score, the system categorizes each exam into three categories representing the probability and risk that a visible cancer is present on the image: low, intermediate, and elevated.

### Control group (without AI support)

In order to compare the performance indicators, a control group from the same CBCSP was collected. This group consisted of consecutive women (age range, 50–69) who were screened with DM or DBT examinations performed with the same two devices, 1 year prior to AI implementation, between 15 March 2020 and 14 March 2021. We excluded recalled women whose diagnostic work-up results were not accessible (Fig. 1). These exams were double read during that period in the screening program by the same team of radiologists as the study group but without artificial intelligence support.

From the available collected cohort, the final selection for the control group was done by statistical matching to ensure balance between control and study group regarding the control variables (Fig. 1).

### Reference standard

Age, type of exam (DM or DBT), breast density, radiological findings, recall indications, biopsy procedures, and histopathologic results were retrieved from the medical records.

We considered cancer those women recalled with histologically confirmed malignancy. And we considered non-cancer those women not recalled and women who were recalled with a benign biopsy result or who were classified as BIRADS 1, 2, or 3 and not submitted to biopsy after the diagnostic work-up performed in the reference hospital.

### Statistical analysis

The first step in the statistical analysis was the implementation of a matching method to ensure balance between control and study group regarding the control variables: modality (DM or DBT), age, and breast density. Nearest Neighbor Matching with Mahalanobis distance was selected as a matching method, and the number of matches was established based on the best performance in terms of balance between the two groups. The last step was the diagnosis of the quality of matches through the standardized mean difference (SMD) of the distance measure as well as the control variables, setting the threshold in 0.1 [28]. The region of common support (that is, overlapping between distributions) was evaluated through density plots and barplots (for continuous and discrete variables, respectively).

The performance of screening in the balanced study and control groups was evaluated and compared in terms of cancer detection rate (CDR), recall rate (RR), and positive predictive value of recalls (PPV). The analysis was done globally and separately for DM and DBT. McNemar test was applied for estimating differences between groups.

All the analyses were carried out with R free software [29]. The packages Machlt [30] and Matching [31] were used for conducting matching analysis, and Imtest [32] and sandwich [33, 34] packages to make inference for estimated logistic regression coefficients.

## Results

### Participant and examination characteristics

From the available 12,011 women in the study group, 11,998 screening examinations from 11,998 women (mean age, 57.59 years ± 5.8 [standard deviation]) were included (99.8%) (5049 digital mammography exams and 6949 digital breast tomosynthesis exams). Thirteen examinations were excluded because the diagnostic workup results were not accessible.

In the control group from the available 16,555 women, 11,998 screening examinations from 11,998 women (mean age 57.94 years ± 5.58 [standard deviation]) were selected after the implementation of a matching method (5045 digital mammography exams and 6953 digital breast tomosynthesis exams) (Fig 1).

Near Neighbor Matching with Mahalanobis distance and a 1:1 ratio without replacement was selected. The standardized mean differences (SMD < 0.1) showed an optimal balance.

The characteristics of the study and control groups are included in Tables 1 and 2.

**Table 1:** Characteristics of study group and control group

Characteristic	Study group	Control group	SMD
Number of women	11,998	11,998	
Mean age at screening (SD)	57.59 (5.80)	57.94 (5.58)	0.062
Mean breast density* (SD)	1.44 (0.78)	1.41 (0.78)	0.041
A+B	6245 (52.0)	6667 (55.6)	
C+D	5753 (47.9)	5331 (44.4)	
Type of exam (mean (SD))	0.42 (0.49)	0.42 (0.49)	0.001
Number of DM	5049 (42.1)	5045 (42.0)	
Number of DBT	6949 (57.9)	6953 (57.9)	

Unless otherwise specified, data are numbers of examinations, with percentages in parentheses

*DBT* digital breast tomosynthesis, *DM* digital mammography, *SD* standard deviation, *SMD* standardized mean differences

^*^Breast density was graded according to the American College of Radiology Breast Imaging Reporting and Data System lexicon

**Table 2:** Summary of cancer characteristics

Characteristic	Study group (*n* = 108)	Control group (*n* = 70)
Morphologic type		
Mass	54 (50)	32 (45.7)
Architectural distortion	20 (18.5)	18 (25.7)
Asymmetry	4 (3.7)	2 (2.9)
Calcifications	30 (27.8)	18 (25.7)
Histologic type		
Invasive ductal carcinoma	74 (68.5)	43 (61.4)
Invasive lobular carcinoma	5 (4.6)	9 (12.9)
Other invasive carcinoma	1 (0.9)	0 (0)
Ductal carcinoma in situ	28 (25.9)	18 (25.7)
Grade		
I	44 (40.7)	26 (37.1)
II	42 (38.8)	25 (35.7)
III	22 (20.3)	19 (27.1)
Size mm (SD)	16.37 (15.8)	20.61 (15.9)

Unless otherwise specified data are numbers of cancers, with percentages in parentheses

*mm* millimeters,* n* number, *SD* standard deviation

### Screening performance with AI system support (study group)

From the total DM and DBT exams, 108 cancers were diagnosed. Cancer detection rate (CDR), recall rate (RR), and positive predictive value of recalled women (PPV1) were 9.0‰ (95% CI: 8.2, 9.7), 6.1% (95% CI: 5.9, 6.3), and 14.6% (95% CI: 13.5, 15.7) respectively (Table 3).

**Table 3:** Comparison of readings with AI system support and readings without AI system in terms of cancer detection rate, recall rate, and positive predictive value

Digital mammography and digital breast tomosynthesis				
Metric	Control group reading without AI	Study group reading with AI system	Difference	*p value*
Cancer detection rate**	5.8 (5.2, 6.4)	9.0 (8.2, 9.7)	+ 3.2 (0.9, 5.4)	< 0.001
Recall rate*	5.4 (5.3, 5.6)	6.1 (5.9, 6.3)	+ 0.7 (0.05, 1.3)	< 0.001
Positive predictive value*	10.6 (9.6, 11.6)	14.6 (13.5, 15.7)	+ 4 (0.3, 7.7)	< 0.001
Digital mammography				
Cancer detection rate**	5.7 (4.8, 6.6)	8.1 (7.0, 9.1)	+ 2.4 (−0.9, 5.6)	0.055
Recall rate*	6.2 (5.9, 6.5)	6.6 (6.3, 6.9)	+ 0.4 (−0.5, 1.4)	0.373
Positive predictive value*	9.2 (7.9, 10.6)	12.2 (10.7, 13.7)	+ 3 (−2.1, 7.9)	0.25
Digital breast tomosynthesis				
Cancer detection rate**	5.8 (5.1, 6.6)	9.6 (8.6, 10.6)	+ 3.8 (0.8, 6.7)	< 0.05
Recall rate*	4.9 (4.7, 5.1)	5.7 (5.5, 6.0)	+ 0.8 (0.01, 1.6)	< 0.05
Positive predictive value*	11.9 (10.4, 13.3)	16.7 (15.2, 18.3)	+ 4.8 (−0.6, 10.2)	0.07

*AI* artificial intelligence

^*^Data are percentages, with 95% CI in parenthesis

^**^Data are rates per 1000 with 95% CI in parenthesis

From the DM exams, CDR was 8.1‰ (95% CI: 7.0, 9.1), RR was 6.6% (95% CI: 6.3, 6.9), and PPV1 was 12.2% (95% CI: 10.7, 13.7) and from the DBT exams CDR, RR, and PPV1 were 9.6‰ (95% CI: 8.6, 10.6), 5.7% (95% CI: 5.5, 6.0), and 16.7% (95% CI: 15.2, 18.3) respectively (Table 3).

**Fig. 2:**
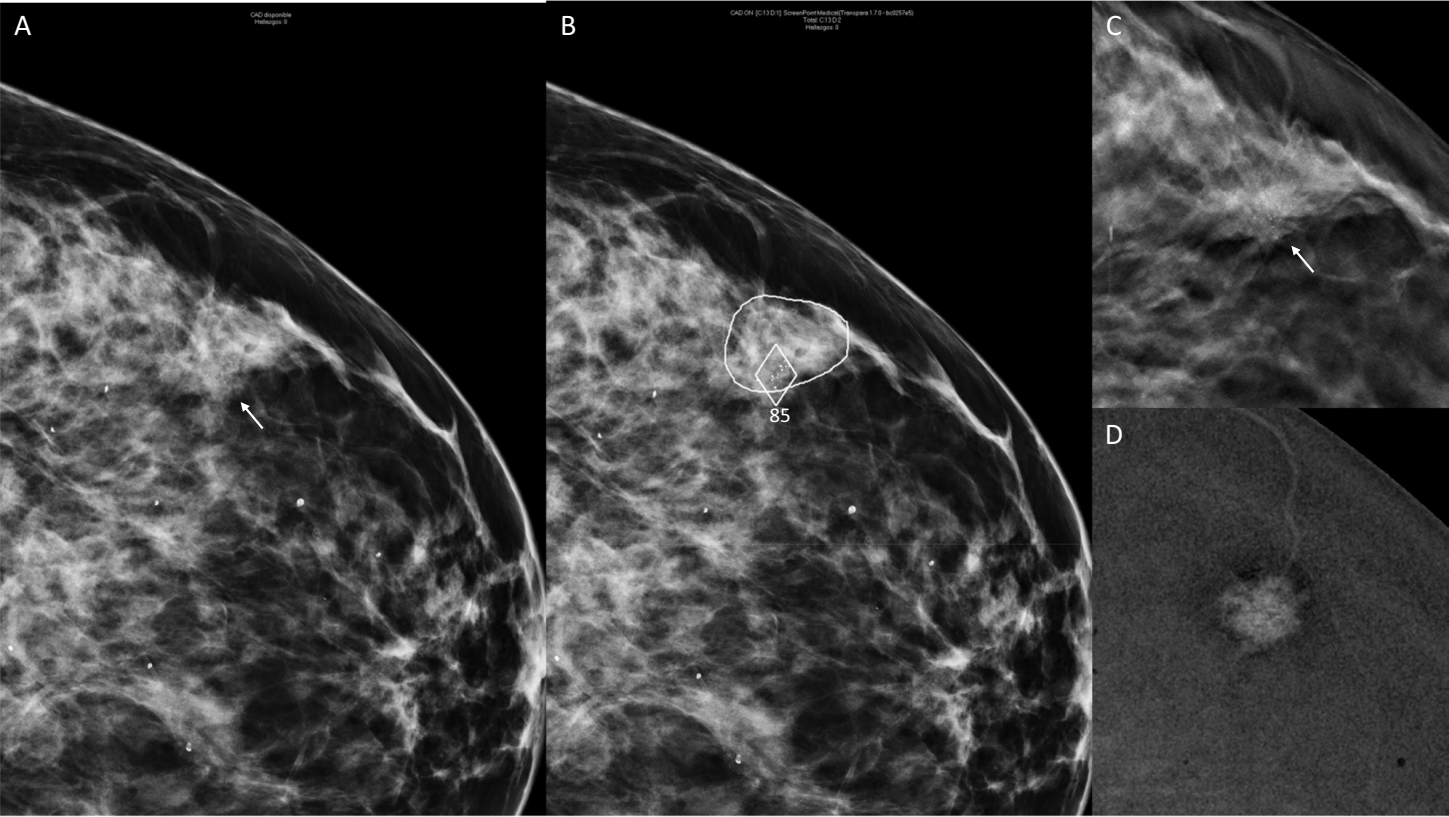
**A** Digital mammography (left craniocaudal view) of a 49-year-old woman, included in the study group, recalled from both readings supported by AI. A subtle asymmetry with calcifications is visible (arrow). **B** Artificial intelligence pinpointed the lesion by circling the asymmetry and marking calcifications with a diamond. It was assigned a score of 85 (elevated risk). **C** During the workload after recalling, a spiculated mass was visible in digital breast tomosynthesis (arrow). **D** Lesion enhanced in contrast enhanced digital mammography. An invasive ductal carcinoma of 15 mm was diagnosed after a US-guided core biopsy

**Fig. 3:**
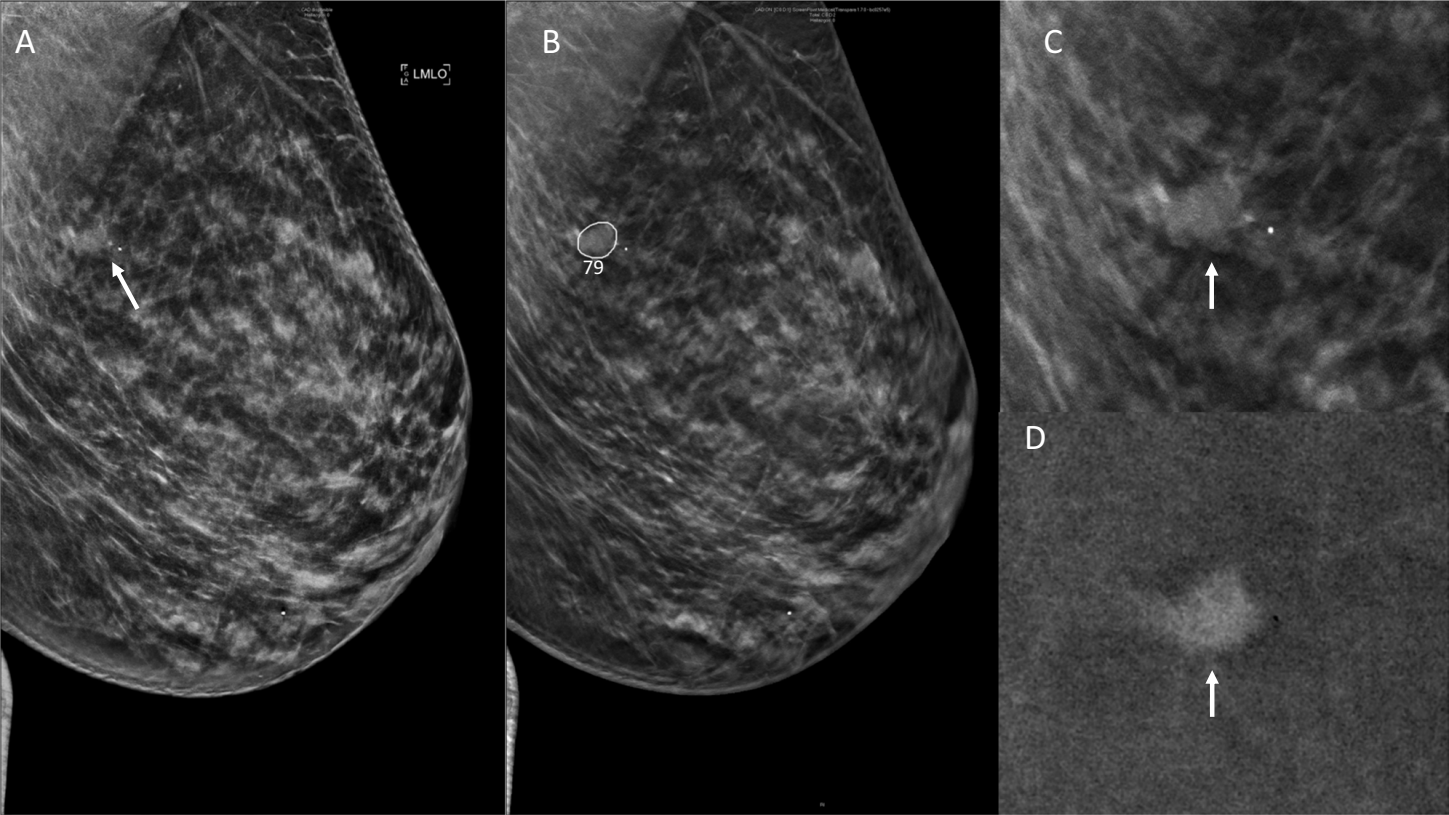
Digital breast tomosynthesis of a 49-year-old woman, included in the study group, recalled from both readings supported by AI. **A** Left mediolateral oblique synthetic (LMLO) view shows a mass (arrow). **B** Digital breast tomosynthesis (LMLO) slice at the level of the lesion. Artificial intelligence circled the lesion and assigned a score of 79 (elevated risk). **C** Spot view of tomosynthesis shows an irregular ill-defined mass (arrow). **D** Lesion enhanced in contrast enhanced digital mammography. An invasive ductal carcinoma of 18mm was diagnosed after a US-guided core biopsy

Illustrative examples of examinations in the study group categorized as elevated risk where the AI system–assisted radiologists in the detection of cancer are shown in Figs. 2 and 3.

### Screening performance without AI system support (control group)

From the total 11,998 DM and DBT exams, 70 cancers were diagnosed. Cancer detection rate (CDR), recall rate (RR), and positive predictive value of recalls (PPV1) were 5.8‰ (95% CI: 5.2, 6.4), 5.4% (95% CI: 5.3, 5.6), and 10.6% (95% CI: 9.6, 11.6) respectively (Table 3).

From the DM exams, CDR was 5.7‰ (95% CI: 4.8, 6.6), RR was 6.2% (95% CI: 5.9, 6.5), and PPV of recalled women was 9.2% (95% CI: 7.9, 10.6) and from the DBT exams CDR, RR, and PPV of recalls were 5.8‰ (95% CI: 5.1, 6.6), 4.9% (95% CI: 4.7, 5.1), and 11.9% (95% CI: 10.4, 13.3) respectively (Table 3).

### Comparison between study and control group

When comparing the global results of reading in the study group with AI system support to the control group without AI, an increase in CDR of 3.2‰ (95% CI: 0–9, 5.4; *p *< 0.001), an increase in PPV of recalls of 4% (95% CI: 0.3, 7.7; *p *< 0.001), and also an increase in RR of 0.7% (95% CI: 0.05, 1.3; *p *< 0.001) was observed (Table 3).

Similar results were obtained in the independent assessment of DM and DBT, although the differences were slightly higher for DBT: an increase in CDR, RR, and PPV1 in the study group was observed compared to the control group of 3.8‰ (*p *< 0.05), 0.8 (*p *< 0.05), and 4.8% (*p *= 0.07) respectively for DBT, and 2.4‰ (*p *= 0.05), 0.4 (*p *= 0.37), and 3% (*p *= 0.25) respectively for DM (Table 3).

### AI standalone performance

**Fig. 4:**
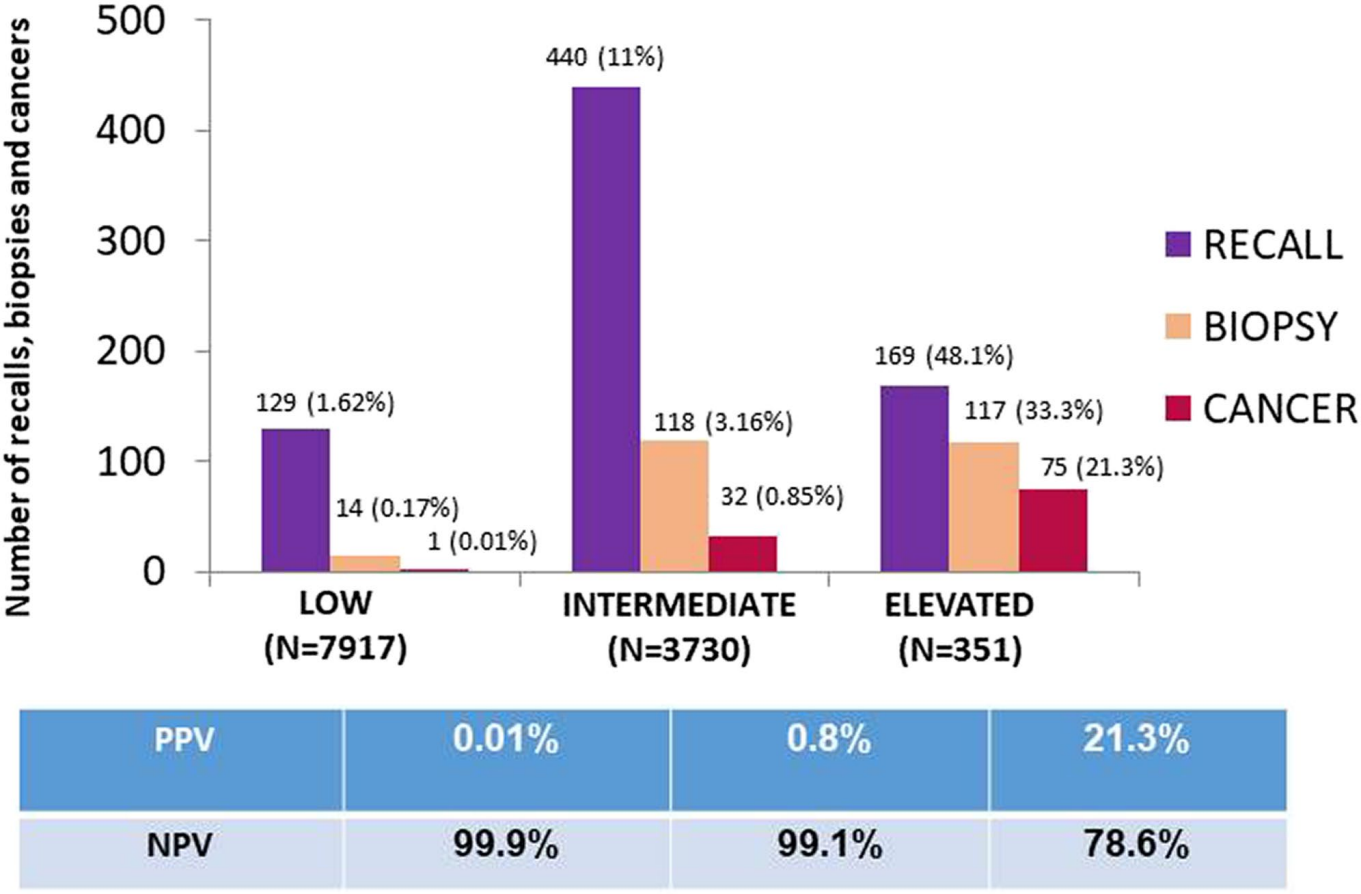
Number of recalls, biopsies, and cancers detected regarding risk categories assigned by the AI ​system in the study group. *N* number, *PPV* positive predictive value, NPV  negative predictive value

The total of the 11,998 included exams in the study group were classified as follows: low, 7917 (65.9%); intermediate, 3730 (31%); and elevated, 351 (2.9%). The 108 studies with cancers were categorized as follows: low, 1 (0.9%); intermediate, 32 (29.6%); elevated, 75 (69.4%) (Fig. 4).

Globally, the probability of cancer in the low- and intermediate-risk categories was less than 2% (0.01 and 0.8% respectively), while in the high-risk category the probability of cancer was 21.3% (Fig. 4).

The AI system correctly marked 101 cancers (93.5%) with the highest score.

## Discussion

In this study, we evaluate the impact in performance of a double reading screening program after the implementation of an AI system for concurrent decision support for the radiologists. The implementation of an AI system for concurrent decision support for reading DM and DBT screening exams increased the breast cancer detection rate (CDR) by 3.2‰ (9.0‰ vs 5.8‰; *p *< 0.001) and the positive predictive value (PPV) of the recalled women by 4% (14.6% vs 10.6%; *p *< 0.001) with an acceptable increase in recall rate (RR) by 0.7% (6.1% vs 5.4%; *p *< 0.001). AI-based performance benefits with respect to CDR and PPV were present when reading DM and DBT studies with AI support. Yet, effects were more pronounced for DBT (CDR + 3.8; PPV of recalled women +4.8; and recall rate + 0.8 in the study group).

Several retrospective studies have shown that radiologists improved their cancer detection performance when using an AI system as a concurrent reading aid. So, reading DM tests with the help of AI was associated with an increase in AUC of between 0.02 and 0.07, as well as an increase in sensitivity and specificity [23, 24, 26]. Similar effects have been reported for DBT. Simultaneous AI support in reading screening tests for DBT resulted in an increase in AUC between 0.03 and 0.057, with no change in specificity [22, 25, 27]. However, all these studies were conducted on small cancer-enriched datasets rather than population-based screening data and the transferability of the results to real-world screening settings might be limited. Our study represents the real-life impact of AI as support for human double reading, in a screening program. Regarding recalls, in our study, we found a slight increase in recall rate (+0.8%) that was accompanied by an increase in the PPV of recalled women by 4.8%, indicating that AI did not increase the number of unnecessary recalls but rather enabled radiologists to recall relevant cases. In the Conant et al study, AI support for reading DBT exams resulted in a reduction in recall rate for non-cancers by 7.2% [25].

The current study tests the ability of the AI system to stratify screening exams based on cancer risk. Overall, 66% of screening exams were classified as low risk and there was only one cancer that was falsely allocated by AI to the low risk category. Less than 3% of all cases were classified as high risk and yet, practically 70% of all cancers were among those cases flagged as high risk. These results are similar to those obtained by other authors in several previous retrospective simulated studies which demonstrated that AI-based stratification screening workflows could achieve workload reduction by up to 70% by identifying a significant volume of low-risk studies that could be partially or fully removed from human reading [11, 14–16, 35, 36]. In addition to providing important information to the radiologist to make a well-informed decision, the AI system’s ability to stratify studies opens the possibility for new strategies in screening programs. Such AI-based screening workflows would reduce the time dedicated to low-risk studies and would allow readers to focus on higher-risk studies that require more attention. Likewise, Dembrower et al [35] demonstrated in a retrospective simulation that the sensitivity of the screening program could be increased if a more intensive screening is carried out for the 1–5% most suspicious exams.

This study has several limitations. First, it has been carried out in a single institution, using two devices from the same vendor and analyzed by only one AI system. Second, the number of false negatives and interval cancers is still unknown since next round cancers and interval cancers remain to be monitored in the future.

In conclusion, this study shows that AI as a concurrent reading aid for human double reading in a real-word screening scenario with DM or DBT significantly increases cancer detection rate and positive predictive value of the recalled women.

## Abbreviations

AI: Artificial intelligence
CDR: Cancer detection rate
DBT: Digital breast tomosynthesis
DM: Digital mammography
NPV: Negative predictive value
PPV: Positive predictive value
RR: Recall rate
SD: Standard deviation
SMD: Standardized mean differences

## Declarations

### Guarantor

The scientific guarantor of this publication is Marina Álvarez Benito.

### Statistics and biometry

María Pata (Biostatech) kindly provided statistical advice for this manuscript.

### Study subjects or cohorts overlap

No

### Methodology

• retrospective

• diagnostic or prognostic study

• performed at one institution
